# Pluripotent stem cell‐derived extracellular vesicles: Cell type‐dependent effect on tumorigenicity in cancer cell lines

**DOI:** 10.1002/ccs3.70017

**Published:** 2026-02-11

**Authors:** Chan Du, Karthikeyan Narayanan, Amudha Ganapathy, Andrew C. A. Wan

**Affiliations:** ^1^ Singapore Institute of Food and Biotechnology Innovation Agency for Science, Technology and Research Singapore Singapore; ^2^ Rensselaer Polytechnic Institute Troy New York USA; ^3^ Department of Oral Biology University of Illinois at Chicago Chicago Illinois USA

**Keywords:** cancer tumorigenicity, drug resistance, extracellular vesicles, human pluripotent stem cells, reprogramming

## Abstract

Extracellular vesicles (EVs) derived from pluripotent stem cells have been reported to reprogram cancer cells to a more benign phenotype, due to its provision of an embryonic microenvironment. Here, we show that the effect of the EVs on the tumorigenicity of the cancer cell lines is cell type‐dependent. First, we characterized the complement of transcription factors contained in EVs derived from human embryonic and induced pluripotent stem cell lines and subsequently treated MCF7, A431, MDA‐MB‐231, and DLD‐1 cancer cell lines with the EVs derived from these pluripotent stem cell lines. For EV‐treated MDA‐MB‐231 and DLD‐1 cells, we found a decrease in the protein expression of CD44 and C24, which are accepted markers for cancer cell populations enriched with tumor‐initiating cells, a result that corresponds to the previous reports. However, for EV‐treated MCF7 and A431 cells, there was an increase in the protein expression of CD44 and C24 instead, with a corresponding increase in clonogenicity and resistance to a panel of anticancer drugs, when compared to non‐exosome‐treated cells. When subcutaneously implanted in nude mice, EV‐treated MCF7 cells gave rise to tumors of larger size than untreated cells. The cell type‐dependence of the effect of hPSC‐derived EV treatment was postulated to be due to occurrence of an EMT for cells located at different locations along the epithelial–mesenchymal spectrum, leading to either an increase or decrease in tumorigenicity. Therefore, exposure to EVs does not always reduce the tumorigenicity of cancer cells but is cell type‐dependent.

## Graphical abstract

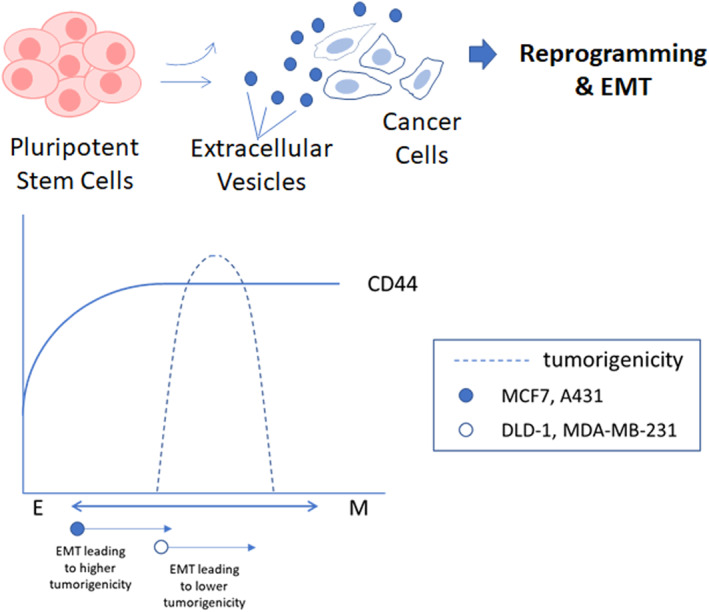

## 1 INTRODUCTION

Extracellular vesicles (EVs) are lipid membrane vesicles that form by outward budding from the plasma membrane, or by fusion of multivesicular bodies within the endosome of eukaryotic cells. These vesicles, of size ranging between ∼40 nm and ∼1 μm in diameter, are secreted by the cells into the extracellular microenvironment, with a suggested role in intercellular communication.^1^, ^2^ EVs from human pluripotent stem cells (hPSCs), which include human embryonic stem cells (hESCs) and induced pluripotent stem cells (hiPSCs), have been reported to contain various proteins, miRNA and mRNA that are involved in the maintenance of pluripotency,^3^ and these same factors are likely to be present in the embryonic microenvironment.

Observations made in pioneering work have shown that the embryonic microenvironment is nonpermissive for tumor development. Cancer cells were epigenetically reprogrammed when implanted into mouse blastocysts and gave rise to normal tissues.^4^, ^5^ Implantation of melanoma cells into mouse embryos lead to their reduced tumorigenicity.^6^ These experiments have in turn led others to investigate the effect of conditioned media derived from embryonic stem cells on cancer cells, or co‐culture of the two cell types, demonstrating further that molecular signals from the embryonic microenvironment can exert an anti‐tumorigenic effect.^7^, ^8^ However, other workers have shown how a cocktail of pluripotent factors can reprogram cancer cells to a tumor‐initiating or cancer stem cell phenotype, which points to a pro‐tumorigenic effect of EVs, considering that they contain many of the same pluripotent factors.^9^

In view of these almost diametrically opposite effects of EVs on cancer cells, we carried out the current work to throw light on the previous observations. Here, we derived exosomes from hESC lines (H1, H7, and HUES7) and one pluripotent stem cell line derived from IMR90 fibroblasts, characterized them for their relevant protein and mRNA content, and investigated their effect on four cancer cell lines in terms of their surface expression of markers for tumor‐initiating cells. Following that, selected EV‐treated cancer cells were characterized in terms of clonogenicity, drug resistance, and in vivo tumorigenicity in a nude mice model.

## 2 MATERIALS AND METHODS

### 2.1 Cell culture

MDA‐MB‐231, MCF7, BT474, and DLD cells were obtained from American Type Culture Collection (ATCC), VA, USA. Cells were cultured at 37°C with 5% CO_2_ in Dulbecco's Modified Eagle's Medium (DMEM) containing 4.5 mg/mL glucose, supplemented with 10% heat inactivated fetal bovine serum, 2 mM L‐glutamine, 50 μg/mL penicillin, and 50 μg/mL streptomycin. Cultures at ∼80% confluence were routinely passaged using trypsin.

### 2.2 Pluripotent stem cell culture

Three hESC lines H1, H7, and HUES‐7 and 1 hiPSC stem cell line (IMR90 derived) were used in the study. The HUES‐7 cell line was obtained from Harvard University (MA, USA). Other cell lines H1, H7, and IMR90‐iPSC were purchased from WiCell Research Institute (WI, USA). Pluripotent stem cells were cultured on a feeder‐free system. Matrigel (BD Biosciences, USA)‐coated plates were used for the culture of the pluripotent stem cells with mTesR1 media. Media was changed every 24 h. Unwanted differentiated cells were physically removed by scraping with a Pasteur pipette. Dispase was used to subculture the cells. Conditioned media were collected from cells grown to a confluence between 60% and 70% every day for 5 days and processed for exosome isolation.

### 2.3 Isolation of EVs from hPSC‐conditioned medium

The hPSCs were cultured on 150 mm (diameter) petri dishes coated with Matrigel as mentioned earlier. Upon achieving 70% confluence, conditioned media were collected for EV isolation. The collected media were cleared of cellular debris by a brief centrifugation at 2800 × g for 20 min. The obtained supernatant was subjected to further centrifugation at 100,000 × g for 70 min. The EV pellet was washed with PBS and collected by centrifugation at 100,000 × g for 70 min. All the centrifugations were performed at 4°C. EVs suspended in PBS were stored at 4°C until further use.

### 2.4 Particle size measurement

Particle size analysis and quantification of exosome concentration was carried out on a ZetaView^®^ Nanoparticle Tracking Analyzer (Particle Metrix GmbH, Inning am Ammersee, Germany). EVs in PBS were observed with blue laser (405 nm), and their Brownian motion was captured for 1 min. A standard detection threshold of 3 with camera level at 14 was used for all measurements. The particle size measurements were performed in triplicates.

### 2.5 Immunoblotting of exosome proteins

Proteins extracted from hPSC‐derived EVs were used for immunoblotting. We performed dot blots to examine protein molecules involved in the cellular reprogramming process. Additionally, western blotting was used to characterize exosome marker proteins.

### 2.6 EV labeling and uptake studies

Isolated exosomes were labeled with membrane‐targeted fluorescent dye PKH67 (Sigma‐Aldrich, St. Louis, MO) and used in the cellular uptake studies. Briefly, 1 × 10^7^/mL EVs in a serum‐free medium was added to cells, and images were visualized at different time intervals using a fluorescence microscope. Uptake studies were performed in triplicates.

### 2.7 RNA isolation and quantitative PCR

Total RNA isolated from EVs and cells using TRIzol reagent (Invitrogen) was reverse transcribed using high capacity cDNA reverse transcription kit (Applied Biosystems, Foster City, CA). Real‐time PCR was carried out in triplicates, in the presence of gene specific TaqMan assays (Applied Biosystems). The RNA copy number was calculated using a standard concentration of respective genes. Primers used are listed in the Table S1.

### 2.8 Reprogramming of cancer cells with exosomes

EVs isolated from 3 pluripotent stem cell sources—two embryonic stem cell lines (H1 and HUES7) and iPSCs (IMR90) were used in the reprogramming of cancer cells. Cancer cell lines used in the EV reprogramming experiments were the breast cancer lines, MCF7 and MDA‐MB‐231, the colon carcinoma line, DLD‐1, and the epidermoid carcinoma line, A431. Each of these cell lines was cultured in the appropriate media, according to suppliers' instructions. The cells were seeded onto 12‐well tissue culture plates at a density of 1 × 10^5^ cells per well and allowed to adhere overnight. Each well was then treated with a 300‐µL medium containing ∼5 × 10^7^ EVs for 3 days. At indicated time points, cells were washed twice with PBS and collected for analysis.

### 2.9 Flow cytometry

EV‐treated hPSCs were dissociated with Accutase™ for 10 min at 37°C, rinsed with PBS, and titurated gently to obtain single‐cell suspension. The cells were processed for staining with antibodies including a primary antibody against CD24 and CD44 followed by Alexa‐Fluor‐488/568‐tagged secondary antibodies. The stained cells were analyzed using a BD LSR II flow cytometer (LSR II, BD, USA).

### 2.10 Colony‐forming assays

MCF7 and A431 cells were cultured for a period of 5 days prior to harvesting for the colony‐forming assay. The cells were resuspended in an agar solution formulated with 1 × DMEM and 0.7% agar (top agar) and layered over the base agar in a petri dish (5000 cells per petri dish; to prevent cells from growing as a monolayer beneath the top agar, a base agar had been first formulated with 1 × DMEM and 0.5% agar, and then coated onto the plate). The plates were placed in an incubator at 37° C for 3 weeks. To visualize the colonies under a stereomicroscope, the cells were stained for an hour with 0.005% crystal violet (Sigma, USA), and quantified.

### 2.11 Drug‐resistance assays

HUES7 EV‐treated and non‐treated A431 and MCF7 cancer cells were seeded at a density of 3 × 10^3^ cells per well in a 96‐well plate, in their respective media. The anticancer drugs, cisplatin, doxorubicin, and sunitinib were then added in concentration series ranging from 0.1 to 1000, 0.001 to 10, and 0.1–1000 μg/mL, respectively. After time periods of 24, 48, and 72 h, the cells were subjected to an Alamar Blue assay for cell viability. From the cell viability–drug concentration curves, corresponding values of IC50 were calculated for a drug exposure period of 24 h, by carrying out linear regression over the range of drug concentration values.

### 2.12 In vivo tumorigenicity assay

The animal studies were conducted according to Agency for Science, Technology and Research (A*STAR) Biological Resource Center IACUC guidelines (Protocol No. #171291) A 100‐μL suspension of 10^8^ MCF7 cells, reprogrammed using EVs from the HUES7 embryonic stem cell line, was injected into the flank of nude mice. Cells that were not treated with EVs were injected in another set of mice, to serve as the controls (*n* ≥ 3). Palpable tumors had formed within 4 weeks, and the sizes of the tumors were monitored every week for the subsequent weeks. Tumor growth was monitored every week by measuring the maximum (L) and minimum (W) length of the tumor using a vernier caliper, following which the tumor volume was calculated by the following formula^10^:

VT=1/2(LxWxW)

## 3 RESULTS

### 3.1 Reprogramming of cancer cells by EV treatment

**FIGURE 1:**
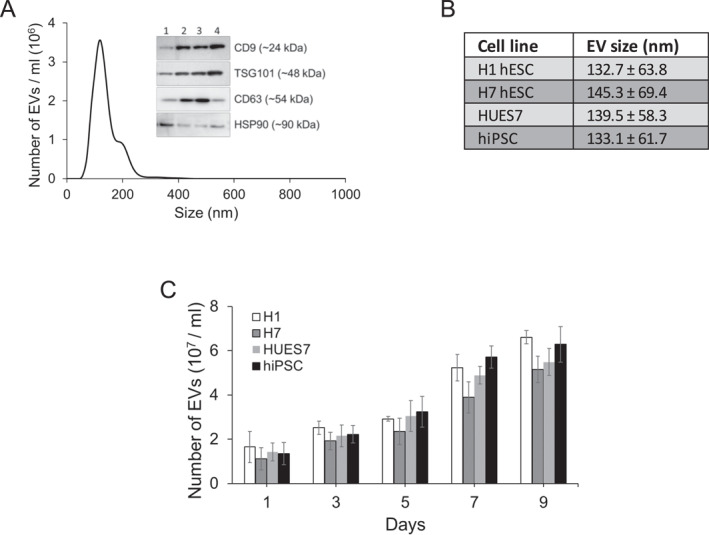
(A) Typical size distribution profile of extracellular vesicles (EVs) derived from hPSC, represented by the HUES7 cell line; inset shows western blot of key EV markers CD9, TSG101, and CD63 in EVs from different hPSC lines. Lane 1: IMR 90 whole cell lysate, Lane 2: IMR90‐IPS EV, Lane 3: HUES7 EV, Lane 4: H1 EV. (B) Average particle size of EVs derived from different hPSC lines. (C) Quantification of EVs over 9 days of culture in different hPSC lines. EVs: extracellular vesicles; hPSC: human pluripotent stem cell.

EVs were extracted from one iPSC line (IMR90) and two hESC lines, H1 and HUES7, and their sizes were measured by dynamic light scattering. The size distribution profile of EVs is represented by the NanoSight graph in Figure 1A. A western blot comparing the key EV markers CD9, TSG101, and CD63 in EVs from the different pluripotent cell lines showed that expression of these markers was higher in the EV fractions than the whole cell lysate, validating the isolation of the EV fraction (Figure 1A). In general, the EVs isolated from these pluripotent stem cells were measured to be between 133 and 145 nm in size (Figure 1B). The number of EVs isolated from multiple cell lines showed a steady increase over 9 days of culture (Figure 1C).

**FIGURE 2:**
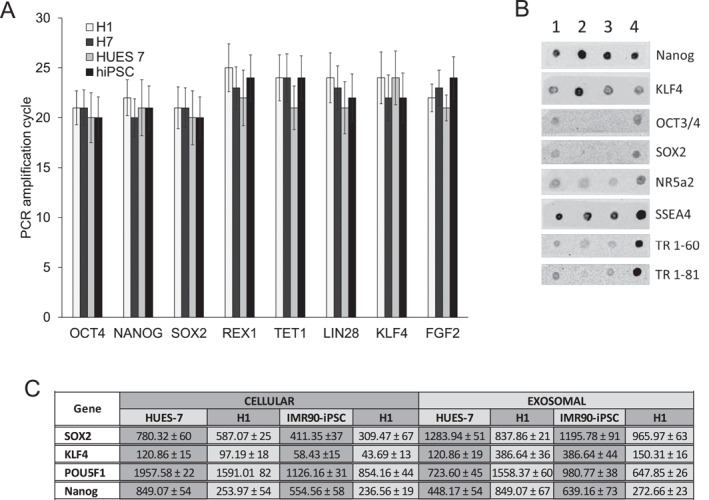
(A) Relative expression of key pluripotent mRNAs in EVs isolated from different pluripotent stem cell sources. (B) Dot blot analysis of protein molecules associated with reprogramming in EVs; Lane 1 = IMR90‐iPS, Lane 2 = IMR90‐IPS EV, Lane 3 = H1 EV, and Lane 4 = HUESs7 EV. (C) RNA copy number of key pluripotent markers. Cellular levels were compared to exosomal levels. EVs: extracellular vesicles.

The presence of pluripotent factors in the form of RNA, proteins, and miRNA has been reported in exosomes derived from pluripotent stem cells. We examined the exosomes for the presence of several RNA species that have been reported to induce the reprogramming of somatic cells.^11^ The relative expression of RNA in EVs from the pluripotent cells were quantitated by real‐time PCR (Figure 2A). Experimental conditions among the EV group (iPSC‐EV, H1‐EV, H7‐EV, and HUES7‐EV) were kept identical. Several protein molecules associated with reprogramming were also expressed, which we examined by dot blot analysis (Figure 2B). EVs isolated from different pluripotent stem cell sources exhibited differences in the expression levels of reprogramming proteins despite similar culture conditions. By comparing the expression of key pluripotent markers in EVs of different cell lines to cellular levels (Figure 2C), two main observations could be made: (a) EVs are selectively higher in some pluripotent RNAs. For example, EVs from all the cell lines are higher in SOX2 and KL4 than the cellular levels and (b) EVs show different levels of expression of the pluripotent RNAs, depending on the cell line they are derived from. Work by other authors have shown that many RNAs are either enriched or excluded from EVs relative to the cells of origin, indicating their selective incorporation into EVs.^12^, ^13^, ^14^, ^15^ Explanations for enrichment of certain types of mRNA, such as cell cycle dependency and zip‐code sequences, have been put forth by other workers.^16^

**FIGURE 3:**
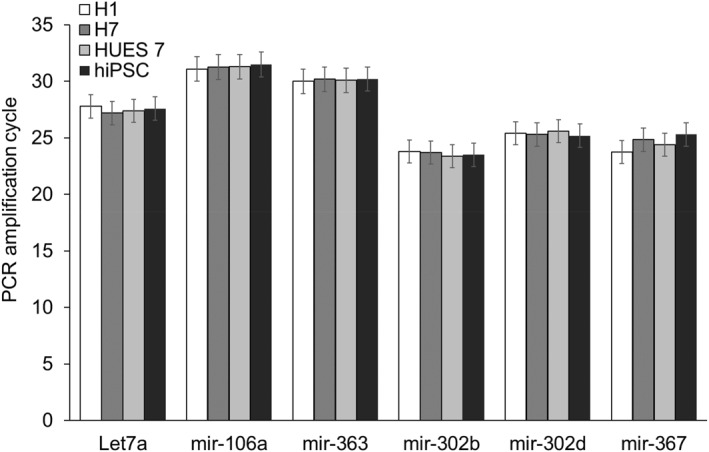
Pluripotent‐related microRNAs expression in extracellular vesicles isolated from different cell lines.

**FIGURE 4:**
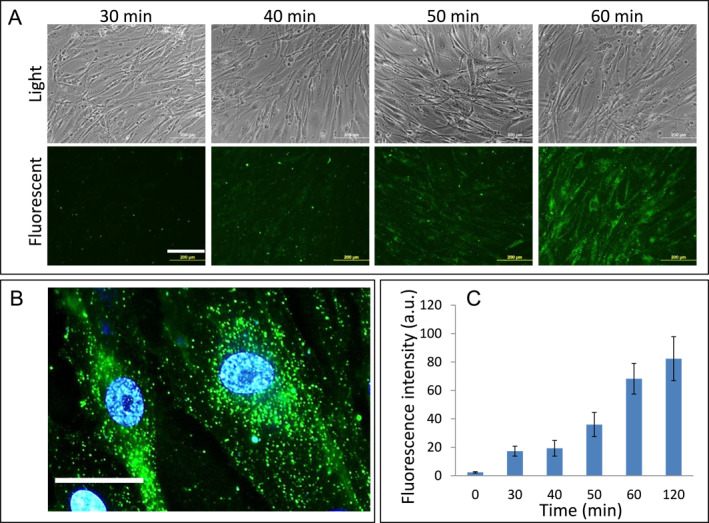
(A) Fluorescence microscopy showing the uptake of labeled EVs by human fibroblasts; scale bar = 200 μm (B) at higher magnification and scale bar = 50 μm (C). Fluorescence intensity measurement of EV uptake. EVs: extracellular vesicles.

Next, we examined the profile of miRNA present in the EVs. miRNAs play a critical role in gene regulation and reprogramming. Earlier reports have identified a set of miRNAs that are involved in reprogramming.^17^, ^18^ We checked for the presence of specific miRNAs in the EVs by real‐time PCR, whereupon we were able to detect Let7a, mir‐125b, mir‐145, mir‐182, mir‐302b, mir‐302d, and mir‐367 (Figure 3). To test the uptake of the EVs, we fluorescently labeled them using PKH26 cell membrane labeling and monitored the kinetics of their uptake in human fibroblasts (Figure 4A). The fluorescently labeled EVs were taken up by the fibroblasts, higher magnification fluorescence images showing the presence of labeled EVs inside the cells, and more particularly, near the perinuclear region (Figure 4B). Quantitative fluorescence intensity measurement indicated that the intensity reached a plateau after 120 min of incubation (Figure 4C).

**FIGURE 5:**
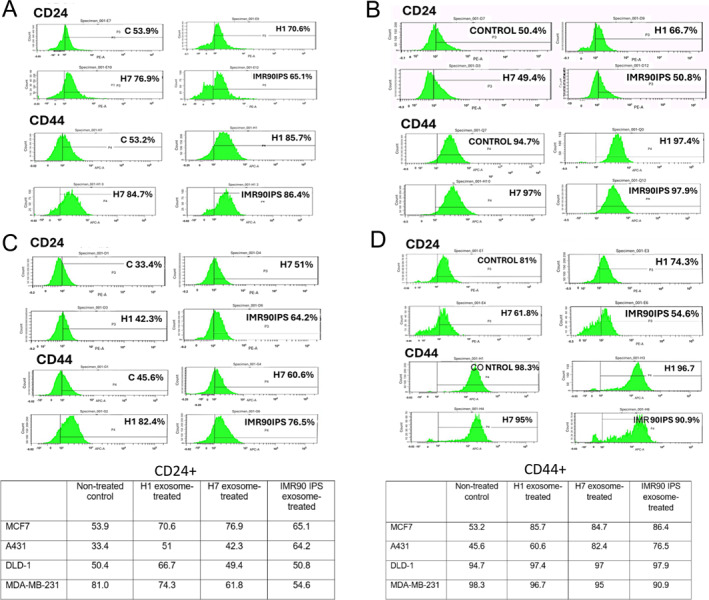
Flow cytometry profiles for CD24 and CD44 expression from 4 cancer cell lines treated with extracellular vesicles derived from human pluripotent stem cells, as compared to untreated control cells (A) MCF7, (B) A431, (C) DLD‐1, and (D) MDA‐MB‐231. The results are summarized in the tables.

From the above experiments, we expected that exposure of the cancer cell lines to the EVs would result in the uptake and subsequent delivery of the reprogramming factors into the cytoplasm. To test whether the EV‐treated cells were enriched in properties of tumor‐initiating cells, we determined the activity of specific cancer stem cell (CSC)‐related surface markers CD24 and CD44 by flow cytometry, with the results summarized in the table (Figure 5). Flow cytometry analysis showed higher CD44 and CD24 expression by the EV‐treated MCF7 and A431 cancer cells, but not the MDA and DLD cell lines. For the MCF7 and A431 cell lines, higher CD44 and CD24 activity was observed for cells subjected to treatment with all EV groups. For the MDA and DLD cell lines, CD44 and CD24 expression were either slightly decreased, or exhibited no significant changes upon EV treatment, with the exception of a higher proportion of CD 24 positive cells for the H1 EV‐treated DLD cells. Thus, MCF7 and A431 cells were used in the subsequent experiments in conjunction with EVs derived from HUES7 and hiPSC cells, which gave the highest levels of CD44 and CD24 upregulation.

**FIGURE 6:**
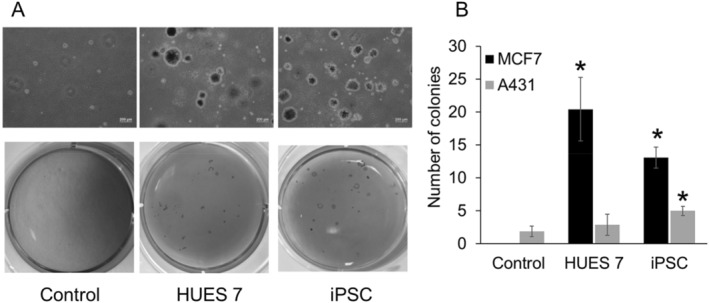
(A) Colony‐forming assay for EV‐treated MCF7 cancer cells. (B) The number of colonies formed by MCF7 cells when treated with HUES7 and hiPSC‐derived EVs, compared to non‐treated control. (C) The number of colonies formed by A431 cells when treated with HUES7 and hiPSC‐derived EVs, compared to non‐treated control. (*n* = 3,*p < 0.01). EVs: extracellular vesicles.

A colony‐formation assay on soft agar was performed to evaluate self‐renewal ability of HUES7 and hiPSC‐derived EV‐treated cells (Figure 6). Treatment with EVs from hiPSC significantly increased the number of colony‐like cell clusters for both the MCF7 and A431 cancer cell lines, while treatment with HUES7‐derived EVs increased the clonogenicity of the MCF7, but not the A431 cancer cell line.

### 3.2 Drug resistance of EX‐iCSCs

**FIGURE 7:**
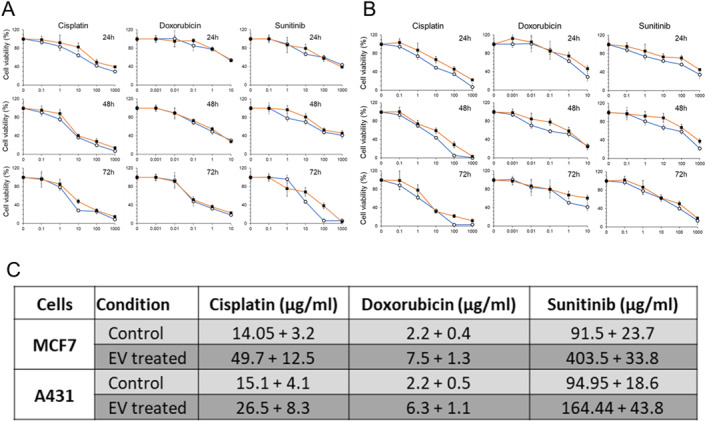
Cell viability of the extracellular vesicle‐treated (A) A431 cancer cell line and (B) the MCF7 cancer cell line, when treated with the cancer drugs, cisplatin, doxorubicin, and sunitinib, respectively. Horizontal axes represent the drug concentrations in μg/mL (*n* = 3). IC50 values for toxicity of the drug to MCF7 and A431 cell lines for a drug exposure period of 24 h.

Drug resistance has been associated with stem cell‐like traits in cancer cells. We treated the MCF7 and A431 cell lines with HUES7‐derived EVs, following which they were subjected to viability (Alamar Blue) assays (Figure 7A,B). EV‐treated cells exhibited significantly higher viability at all time points especially for EV‐treated A431 cells exposed to Sunitinib, and EV‐treated MCF7 cells exposed to doxorubicin. From the cell viability–drug concentration curves, corresponding values of IC50 were calculated for a drug exposure period of 24 h, following the methodology described in Figure S1. The EV‐treated samples were observed to exhibit higher values of IC50, indicating a conferred resistance to the drug (Figure 7C).

### 3.3 In vivo tumorigenic assay

From the results of the clonogenicity assay, treatment of MCF7 cells with EVs derived from the HUES7 embryonic stem cell line gave rise to the largest difference in the number of colonies between treated and non‐treated cells. Thus, the same conditions were employed to obtain reprogrammed cancer cells that were implanted subcutaneously into nude mice.

**FIGURE 8:**
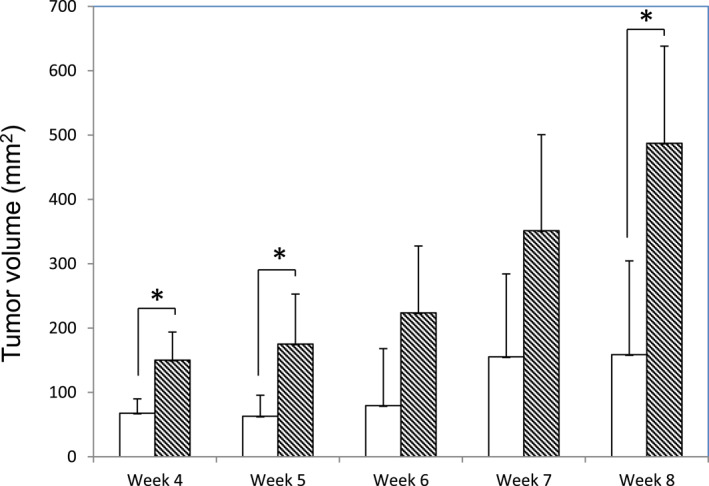
In vivo tumorigenic assay. EV‐reprogrammed MCF7 cells were injected into the flank of nude mice. Palpable tumors had formed within 4 weeks, and the size of the tumors were monitored every week for the subsequent 4 weeks. White bars: non‐treated controls and black hatched bars: EV‐treated samples. (*n* ≥ 3). EV: extracellular vesicle.

For almost all of the measured time points (4–8 weeks), EV‐treated cells led to tumor masses of larger dimensions than the untreated controls (Figure 8).

### 3.4 Starting EMT status of cell lines and changes upon exosome treatment

From the flow cytometry results of Figure 5, we could derive information on the starting EMT status of the cell lines. Besides being a marker for cancer stem cells, CD44 is also known to be a mesenchymal marker.^19^ CD44 is positively correlated with mesenchymal genes such as SLUG, SNAI1, ZEB1, and TWIST1 and negatively correlated with epithelial markers such as E‐cadherin.^20^ Our experiments indicated that MCF7 and A431 are more epithelial in nature, with lower % of CD44 (53.2% and 45.6%, respectively), while the higher levels of CD44 for the DLD‐1 and MDA‐MB‐231 cell lines (94.7% and 98.3%, respectively) indicated their mesenchymal phenotype.

**FIGURE 9:**
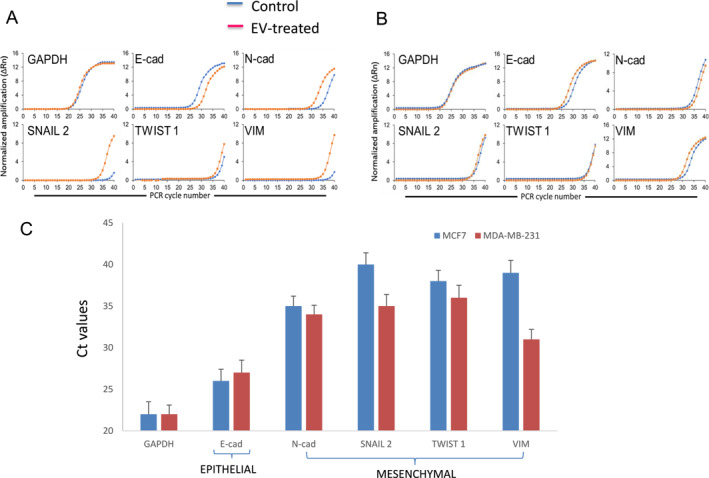
Key EMT gene expression analysis in (A) MCF7 and (B) MDA231 cells. Data show the PCR amplification curve (lower cycles and higher cycles represent higher expression and lower expression, respectively); (C) Ct values for non‐treated (control) MCF7 and MDA‐MB‐231 cells, plotted in the form of a bar graph, with error bars representing mean +standard deviation for PCR assay triplicates. Lower Ct values indicate higher expression of the gene. Results indicate the starting EMT status of MCF7 cells to be epithelial and MDA‐MB‐231 cells to be mesenchymal.

To further confirm the starting EMT status of the cell lines and changes in status upon exosome treatment, the MCF7 and MDA‐MB‐231 cells were treated with exosomes derived from HUES7 cells. The resulting gene expression (qPCR) profiles for several EMT markers, for both control and exosome treated cells, are shown in Figure 9A,B. There was a decrease in the gene expression of ECAD and increases in N‐cad, Snail2, Twist 1, and Vim for the MCF7 cell line. In general, however, there were only slight changes in expression of the same for the MDA‐MB‐231 cell line. Using Ct values, the starting EMT status for MCF7 and MDA‐MB‐231 with respect to these selected EMT markers were also compared (Figure 9C).

## 4 DISCUSSION

Previous studies have shown that cancer cells can be reprogrammed to a benign phenotype, or even give rise to normal tissue, in an embryonic microenvironment.^4^, ^5^ More recently, this has been followed up by work that demonstrated the antiproliferative and proapoptotic effects of hPSC‐derived EVs on cancer cells.^21^ In related work, cancer cells have also been reprogrammed with factors that can return them to a pluripotent or a partially pluripotent state, and subsequently differentiated to cells of the various germ layers.^22^, ^23^, ^24^, ^25^ However, there is a large body of work showing that EVs derived from other stem cell types, in particular mesenchymal stem cells, can promote cancer cell proliferation, migration, and/or invasiveness, with contradictory evidence that suggests their role in suppressing tumors.^26^ The content of pluripotent factors in the form of proteins or RNA, in pluripotent cell‐derived EVs, also indicate that reprogramming cancer cells with the latter may result in enhanced stemness characteristics, which is often associated with greater malignancy. This led us to reexamine the effect of EVs on the tumorigenicity of cancer cells.

In our work, we observed that hPSC‐derived exosomes enhanced the expression of the tumor‐initiating cell markers, CD24 and CD44,^27^ of the cancer cell lines MCF7 and A431. Conversely, there was either no significant change or generally, a slight reduction of these markers for the DLD‐1 and MDA‐MB‐231 cells that were exposed to the EVs. As the apparent increase in fraction of tumor‐initiating cells for MCF7 and A431 indicated an opposite effect of EV treatment from what has been reported in literature,^7^ we went on to characterize their colony‐forming ability and tumor formation in vivo, confirming that the tumorigenicity of these EV‐treated cells was indeed enhanced.

Clearly, there is a cell type‐dependence in the effect of pluripotent cell‐derived EVs on the tumorigenicity of different cancer cell lines, and we hereby postulate a mechanism that accounts for this cell type‐dependence. In a paper from the Weinberg lab,^28^ it was shown that the tumor‐initiating ability of cancer cells was highest when the cells adopted a phenotype that was intermediate, in between epithelial and mesenchymal states. This is illustrated in Figure S2a, with tumorigenicity indicated by the dotted line. MCF7 and A431 are more epithelial in nature, with lower % of the mesenchymal stem cell marker, CD44 (53.2% and 45.6%, respectively). On the other hand, DLD‐1 and MDA‐MB‐231 possess a more mesenchymal phenotype, expressing high levels of CD44 (94.7% and 98.3%, respectively). The representative positions of these cancer cell lines along the epithelial (E)–mesenchymal (M) axis have been indicated. It is hypothesized that the tumorigenicity of DLD‐1 and MDA‐MB‐231 are already close to the peak (of the dotted line); thus, any transition toward the mesenchymal phenotype, as indicated by the single arrows, would lead to a drop in tumorigenicity. Conversely, for the MCF7 and A431 cell lines, transition toward the mesenchymal phenotype would lead to an increase in tumorigenicity.

For the above proposed mechanism to work, exposure of the cancer cell lines to the EVs would have to result in a transition toward the mesenchymal state. Using the STRING database, we constructed the interactions between some of the miRNA and RNA present in the EVs (Figure S2b). Indeed, these interactions reveal how the miRNA and RNA molecules can promote EMT. Furthermore, we showed experimentally that the EMT‐promoting effect only occurred for MCF7 cells (epithelial phenotype) and not MDA‐MB‐231 cells (mesenchymal phenotype) (Figure 9A,B). When treated with EVs, only the MCF7 cells and not the MDA‐MB‐231 cells, showed a substantial decrease in gene expression of the epithelial marker E‐cad, and increase in gene expression of the mesenchymal markers N‐cad, Snail 2, Twist 2, and Vim. This result correlated well with the starting EMT status of the two cell types, respectively (Figure 9C). Thus, we hypothesize that reprogramming of the cancer cell lines in this work was induced by the reprogramming factors (proteins, RNA, and miRNA) present in the EVs, and accompanied, at least in part, by an EMT.

In short, the cell type‐dependence on induction of tumorigenicity by EVs as demonstrated in this paper correlates well with the emerging concept in the field, where cells with an intermediate, hybrid mesenchymal/epithelial phenotype were found to be more tumorigenic than cells residing in a more complete mesenchymal or epithelial state. The more epithelial MCF7 and A431 cancer cell lines are induced to a more tumorigenic phenotype by gaining mesenchymal characteristics via an EMT, whereas the more mesenchymal DLD‐1 and MDA‐MB‐231 cell lines are unaffected or become less tumorigenic with the EMT, by virtue of moving away from the intermediate phenotype.

Applying the EVs to MCF7 and A431 cells, as presented in this work, can be thought of as a method to induce cancer cells to cancer stem cell‐like cells. Besides the use of EVs to reprogram cancer cells, other methods such as transcription factor reprogramming and conditional reprogramming are available. Transcription factors that direct cell fate in normal cells can act as oncogenes, by rewiring the cells to acquire developmental programs required for tumor formation. Suva et al. were able to reprogram differentiated glioblastoma cells to tumor‐propagating cells with stem cell‐like properties by introducing a set of neurodevelopmental transcription factors.^9^ In conditional reprogramming, a combination of the Rho‐kinase inhibitor and culture of cancer cells on irradiated fibroblast feeder layers leads to transient acquisition of stem‐like properties.^29^ These cells, although a relatively recent development, have already shown their utility for the long‐term culture of primary cancer cells. In the same way EV‐induced cancer cells may find application in drug screening for the identification of drugs that target the cancer stem cell population, despite the stem‐like cells not being exactly identical to the CSC in tumors. Further work to characterize the phenotype of the cancer stem‐like cells generated in the present work, for example, in their expression of drug transporters, would shed more light on their observed drug resistance and similarity to naturally occurring CSCs.

## 5 CONCLUSION

In the present work, we have shown that treatment of specific cancer cell lines with EVs derived from hPSCs can reprogram them to a cancer stem cell‐like phenotype associated with higher clonogenicity, drug resistance, and tumorigenicity. Besides demonstrating that the effect of EV treatment on the tumorigenicity of cancer cell lines is cell type‐dependent, the resulting EV‐treated cells may potentially be useful for anticancer drug screening and the identification of drugs that target cancer stem cells.

## AUTHOR CONTRIBUTIONS

**Chan Du**: Conceptualization; investigation; formal analysis; writing—review and editing. **Karthikeyan Narayanan**: Conceptualization; investigation; formal analysis; writing—review and editing. **Amudha Ganapathy**: Investigation; formal analysis. **Andrew C. A. Wan**: Conceptualization; writing—original draft; writing—review and editing.

## CONFLICT OF INTEREST STATEMENT

The authors declare no conflicts of interest.

## ETHICS STATEMENT

Ethical approval was required for the animal study in this work and obtained from (A*STAR) Biological Resource Center IACUC (Protocol No. #171291).

## Supporting information

Supporting Information S1

## Data Availability

All data are incorporated into the article and its online supplementary material.
