# Supplementary material for: Pluripotent stem cell‐derived extracellular vesicles: Cell type‐dependent effect on tumorigenicity in cancer cell lines
Source: J Cell Commun Signal. 2026 Feb 11;20(1):e70017. doi: 10.1002/ccs3.70017 (PMC12893097; doi:10.1002/ccs3.70017)
Supplement: Supplementary file 1 — Supporting Information S1 [file CCS3-20-e70017-s001.docx]

**Supplementary Information**

**Pluripotent stem cell-derived extracellular vesicles: cell type-dependent effect on tumorigenicity in cancer cell lines**

Chan Du, Karthikeyan Narayanan, Amudha Ganapathy, Andrew C.A. Wan


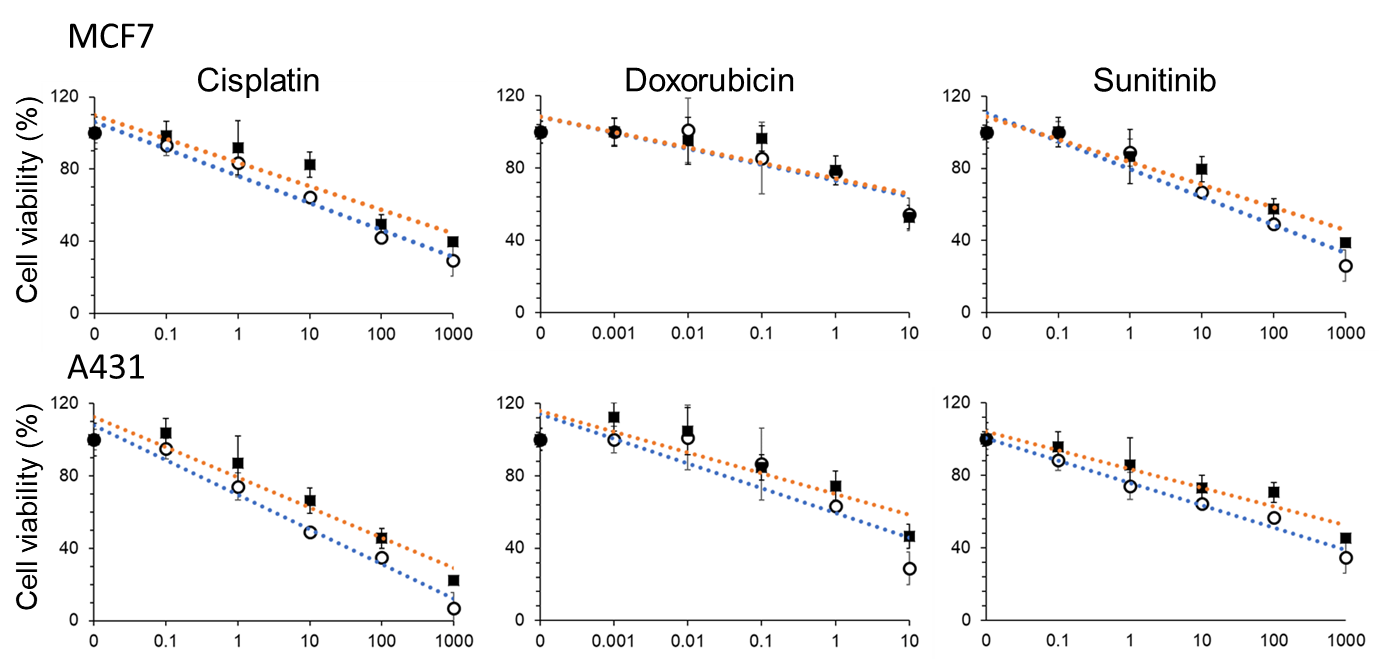


**Figure S1**: The IC50 values for MCF7 and A431 cells exposed to cisplatin, doxorubicin and sunitinib for a period of 24 h were calculated by carrying out linear regression over the range of drug concentration values. Corresponding values of control and EV-treated cells were then obtained for a cell viability of 50%.


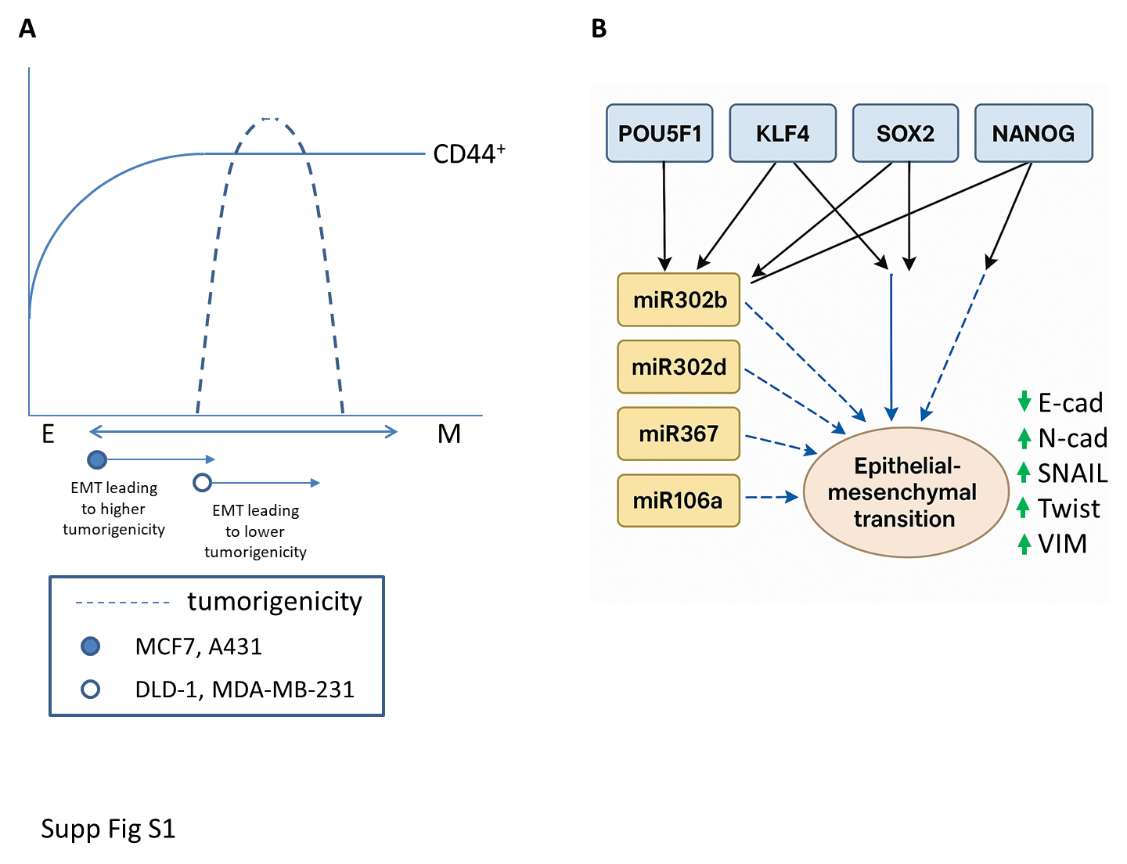


**Figure S2**: (A) Working model representing the transition of cell state (indicated by arrows) for different cancer cell lines along the epithelial (E)-mesenchymal (M) axis, upon reprogramming with EVs. The plot of CD44 level superimposed on the `tumorigenicity’ curve, with an epithelial-mesenchymal horizontal axis, is based on that of Bierie et al. [28]. (B) Interactions between miRNA and RNA in EVs, leading to EMT.

**Table S1**: List of primers for TaqMan assays

mRNA

| SOX2 | Hs00415716_m1 |
| --- | --- |
| KLF4 | Hs00358836_m1 |
| POU5F1 | Hs03005111_g1 |
| Nanog | Hs02387400_g1 |
| REX1 | Hs00399279_m1 |
| TET1 | Hs00419323_m1 |
| LIN28 | Hs01013729_m1 |
| FGF2 | Hs00266645_m1 |

miRNA

| Let7a | 000377 |
| --- | --- |
| mir-106a | 002170 |
| mir-363 | 001283 |
| mir-302b | 002119 |
| mir-302d | 000535 |
| mir-367 | 002121 |

**List of antibodies used**

CD9 (C-4) sc-13118 (Santa Cruz)

CD63 (H-163) sc-15363 (Santa Cruz)

TSG 101 (C-2) sc-7964 (Santa Cruz)

NANOG sc-293121 (Santa Cruz)

OCT-3/4 sc-5279 (Santa Cruz)

SOX 2 sc-365823 (Santa Cruz)

TRA-1-81 sc-21706 (Santa Cruz)

TRA-1-60 sc-21705 (Santa Cruz)

KLF4 (F-8) sc-166238 (Santa Cruz)

SSEA4 MC-813-70 (Sigma Aldrich)

HSP90 ab13495 (Abcam)
